# Th17 Lymphocytes in Respiratory Syncytial Virus Infection

**DOI:** 10.3390/v5030777

**Published:** 2013-03-05

**Authors:** Jonas Bystrom, Nasra Al-Adhoubi, Mohammed Al-Bogami, Ali S. Jawad, Rizgar A. Mageed

**Affiliations:** 1 Bone and Joint Research Unit, William Harvey Research Institute, Queen Mary University of London, London, EC1M 6BQ, UK; E-Mails: m.al-bogami@qmul.ac.uk (M.A.-B.); r.a.mageed@qmul.ac.uk (R.A.M.); 2 Department of Rheumatology, The Royal London Hospital, Mile End, Barts and The London, Queen Mary University of London, London, EC1M 6BQ, UK; E-Mails: nasrak2004@yahoo.com (N.A.-A.); alismjawad1@hotmail.com (A.S.J.)

**Keywords:** RSV, pneumovirus, mucus, interleukin 17, interleukin 23, interleukin 13, Th17

## Abstract

Infection by respiratory syncytial virus (RSV) affects approximately 33 million infants annually worldwide and is a major cause of hospitalizations. Helper T lymphocytes (Th) play a central role in the immune response during such infections. However, Th lymphocytes that produce interleukin 17 (IL-17), known as Th17 lymphocytes, in addition to been protective can also cause pathology that accompany this type of infection. The protective effects of Th17 is associated with better prognosis in most infected individuals but heightened Th17 responses causes inflammation and pathology in others. Studies employing animal models haves shown that activated Th17 lymphocytes recruit neutrophils and facilitate tertiary lymphoid structure development in infected lungs. However, IL-17 also inhibits the ability of CD8^+^ lymphocytes to clear viral particles and acts synergistically with the innate immune system to exacerbate inflammation. Furthermore, IL-17 enhances IL-13 production which, in turn, promotes the activation of Th2 lymphocytes and excessive mucus production. Studies of these animal models have also shown that a lack of, or inadequate, responses by the Th1 subset of T lymphocytes enhances Th17-mediated responses and that this is detrimental during RSV co-infection in experimental asthma. The available evidence, therefore, indicates that Th17 can play contradictory roles during RSV infections. The factors that determine the shift in the balance between beneficial and adverse Th17 mediated effects during RSV infection remains to be determined.

## 1. Th17 lymphocytes and IL-17 and the immune system—basic biology

Th17 lymphocytes play a central role in host defences against a range of extracellular pathogens including bacteria, viruses and fungi [1,2,3]. This subset of helper T lymphocytes differ from the other subsets, Th1, Th2 and regulatory T lymphocytes (T-reg lymphocytes) both in their requirements for differentiation and expansion factors and in their targets pathogens. In addition, excess Th17 lymphocyte numbers have been associated with inflammatory autoimmune diseases [4,5,6]. In contrast to Th1 and Th2 lymphocytes which predominantly produce interferon gamma (IFNγ) and IL-4/IL-5/IL13, Th17 lymphocytes produce IL-17A, IL-17F, IL-21 and IL-22. IL-17A and IL-17F are members of IL-17 family of cytokines which includes six members; IL-17A-F. All members of this family are involved in inflammatory responses; however, only IL-17A, F and E (IL-25) are produced by haematopoietic cells. IL-17A and IL-17F show 50% homology and both bind IL-17 receptor (IL-17R) which is a complex of IL-17RA and IL-17RC. IL-17A binds with higher affinity to the IL-17RA/C and induces stronger intracellular signalling than IL-17F. Both IL-17A and F are active as dimers; homodimers and heterodimers [7]. The studies reviewed in this article are mainly about IL-17A and this cytokine will thereafter be referred to as IL-17. 

IL-17 acts on stromal cells to promote the production chemokines such as CXCL1, IL-8, CCL20 (MIP-3α) and IL-6 which then promote neutrophil recruitment to sites of infection (see Table 1) [8]. IL-17 itself is a weak inducer of these cytokines/chemokines but acts by stabilizing mRNA transcripts induced by other cytokines [9]. IL-17 also down-regulates micro-RNA 23b (miR-23b), which negatively regulates inflammatory responses [10]. Furthermore, IL-17 induces mucus production in the respiratory tract and increases the expression of polymeric Ig receptors that facilitate the release of IgA and IgM antibodies into the respiratory tract [11]. Of the other cytokines produced by Th17 lymphocytes, IL-21 promotes Th17 proliferation and antibody production by B lymphocytes [12]. Paradoxically, however, Il-21 also antagonizes some IL-17-mediated responses during RSV infection [13]. IL-22, in contrast, promotes mucosal homeostasis and induces the production of antibacterial peptides [14].

The production of low-levels of IL-17 by resident Th17 lymphocytes is necessary for maintaining immunological homeostasis in the gut. This occurs under the influence of IL-1β and transforming growth factor beta (TGFβ) that are produced by gut epithelial cells [15]. During inflammation, IL-6 and prostaglandin E_2_ (PGE_2_) are produced and these induce IL-23 receptor expression which is necessary for the differentiation of naive CD4^+^ T lymphocytes to Th17 lymphocytes [16,17]. The differentiation of Th17 cells involves an intricate network of cytokines and transcription factors predominant among which is the retinoic orphan receptor gamma t (RORγt) and retinoic acid receptor alpha (RARα) [18]. Interestingly, recent studies have revealed that low level CD3/TCR engagement, as compared with high level receptor engagement, preferentially promotes human Th17 differentiations and the effect is mediated through activating the NFAT-1 transcription factor [19,20]. Hypoxia also promotes Th17 differentiation through the hypoxia-inducible factor alpha (HIF-1α) transcription factor which binds to the promoter of RORγt in naïve T lymphocytes [21,22]. The production of IL-23, in contrast, is associated with the expansion of Th17 lymphocytes in pathogenic settings such as in autoimmune disease [23]. In this respect, IL-23 production by DCs during RSV infection has been suggested to be responsible for Th17 propagation and exacerbated inflammation associated with the infection [24,25]. 

## 2. Th17 lymphocytes in the respiratory tract

Th17 lymphocytes are present in the respiratory tract and there is evidence that they play a key role in responses to fungal infections. These cells, however, also contribute to inflammatory disorders that afflict the respiratory tract, such as asthma and chronic obstructive pulmonary disease (COPD). Increased production of the Th17-related cytokines, such as IL-17A, IL-22 and IL-23 in COPD patients reflects the involvement of Th17 lymphocytes in initiating and driving the disease process [26,27]. In addition, excess IL-17 production has been reported in animal models and human patients has been associated with neutrophil dominated asthma and with cortisone-resistant severe airway hyper-reactivity (AHR) [28,29]. Although both IL-17A and IL-17F have been shown to play a role in asthma, studies of gene knockout mice have suggested that IL-17F may in fact ameliorate the disease process [30]. Th17 lymphocytes have also been implicated in effector mechanisms triggered in response to RSV and other types of respiratory viral infections [31,32].

## 3. IL-17 and Th17 lymphocytes in human RSV infection

### 3.1. The immune response at the onset of RSV infection

Worldwide, infants are affected by lower respiratory tract infections caused by RSV. Although many such infections have a mild course, in certain infants the infection leads to bronchiolitis needing hospitalization and respiratory support in an intensive care unit [31,34,35]. Inhaled RSV particles bind glucose amino glycans on respiratory epithelial cells through their glycoproteins, major attachment protein G and fusion protein F. The particles then fuse with the cells and initiate their propagation and spreading [36,37]. There is evidence that cells other than epithelial cells, including macrophages and dendritic cells (DCs), are also infected by the virus [38,39]. Infected epithelial cells initially respond either by releasing acute phase proteins or promoting their production, such as causing complement component C3 activation and the release of its pro-anaphylactic factor C3a [40]. The subsequent response to the infection is of innate immune-type resulting in the influx of neutrophils which become the dominant cells during the first four days of infection [41,42]. 

**Figure 1:**
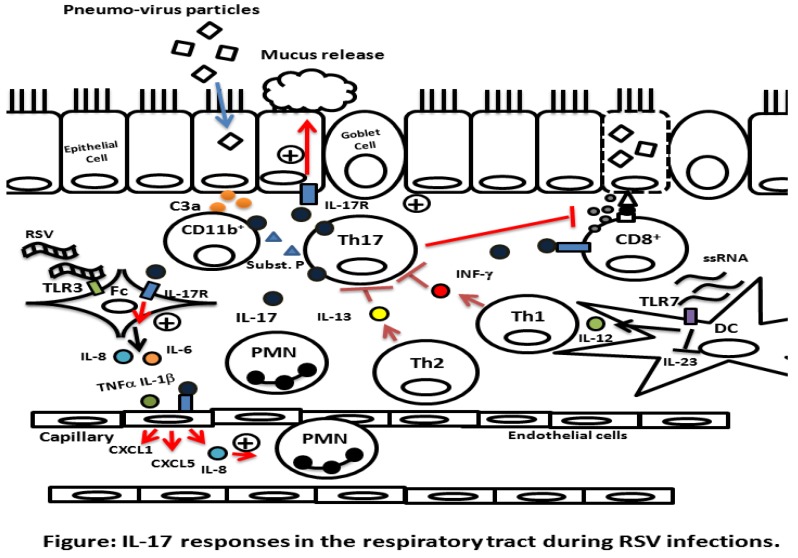
IL-17 mediated responses in the respiratory tract during RSV infections. RSV virus particles infect ciliated epithelial cells in the lower respiratory tract. C3a and other mediators of inflammation are then released from epithelial cells in response to the infection and this, in turn, induces IL-17 production (dark blue colour). During the early phase of the response to RSV infection, IL-17 is produced by CD11b^+^ innate immune cells. Subsequently, the production of IL-17 is predominantly by CD4^+^ Th17 lymphocytes. The production of IL-17 initiates a number of effects in the respiratory tract. Thus, IL-17 induces mild inflammation and exacerbates inflammatory responses triggered by other signals and cytokines. In this scenario, single stranded RNA in RSV particles bind to TLR3 and synergize with IL-17 to induce IL-6 (orange colour) and IL-8 (blue colour) by fibroblasts (Fc). The binding of double stranded RNA to TLR7, however, is inhibitory to IL-17-mediated responses and, instead, promotes Th1-mediated responses. IL-17 co-operates with IL-1β and TNFα to induce the release of chemokines that mediate neutrophil recruitment. Furthermore, IL-17 induces mucus production from epithelial cells. IL-17 also binds receptors on CD8^+^ T lymphocytes and inhibits their ability to reduce viral load. Cytokines produced by Th1 and Th2 lymphocytes, IFNγ and IL-13, in contrast, inhibit IL-17 production [25,33].

### 3.2. The adaptive immune response to RSV infection

RSV infection activates T lymphocytes in lung draining lymph nodes with the help of DCs that migrate from sites of infection. This results in the induction and differentiation of T lymphocytes into viral-specific Th1 and Th2 lymphocytes in detectable numbers in the lung 6-8 days post RSV infection [43], 46]. CD8^+^ T lymphocytes emerge after the initial inflammatory response that follows RSV infection to clear viral particles [42]. Th1 lymphocytes are also induced and these produce pro-inflammatory cytokines, such as IFNγ and TNFα and the combination of the two cellular responses efficiently clears RSV infections in most individuals [44]. However, humans and mice deficient in the transcription factor STAT1, which is activated following IFNγ binding to its receptor, are prone to severe RSV infections [45,46]. In addition to cytotoxic and Th1 lymphocytes, the immune response also includes Th2 lymphocytes which cause key symptomatic features of RSV infections, such as excessive mucus production and wheezing that normally accompany asthma [47]. Measurement of cytokines in the bronchoalveolar lavage (BAL) of infected infants (1.5-6 months of age) has identified similar levels of Th1- and Th2-type cytokines [48], [49]. In animal models of RSV infection, the lack of Th1 effects resulting from IFNγ receptor deletion leads to a dominant Th2 response and worse pathology suggesting that Th1 responses ameliorate Th2-mediated effects during RSV infection in humans [50]. A recent study analysing the cytokine profile in the plasma of RSV infected infants (6 months or younger), however, revealed that infants with a moderate response to the virus had higher plasma levels of IL-17 than infants with a severe response to RSV. In this study IFNγ and TNFα levels were shown to be lower in RSV infected infants than in controls infants [51]. Furthermore, IL-17 levels were higher in BAL from paediatric patients (13 months and below) with non-ventilated RSV disease at admission and at discharge compared with BAL from more severe, ventilated cases. IFNγ was undetectable in this study but IL-6 levels were 30 times higher in the ventilated cases [31]. A further study which examined tracheal aspirates reported increased IL-6 and IL-17 levels in severely ill ventilated infants compared with healthy infants (age not specified in this study) [32]. It is currently unknown what the function of IL-17 is in the respiratory tract and why higher levels are associated with better outcome in some but not all infected infants. One clue could be that the immune system of the newly born is immature with an impaired Th1 response [52]. DCs derived from infants’ umbilical cord blood have, for example, been shown to produce low levels of IL-12 [53]. Furthermore, DCs from cord blood of newly born, but not DCs from the blood of adults, when infected with RSV induced IL-17 production when co-cultured with T lymphocytes [39]. DCs from infected infants were shown to produce TGFβ, a cytokine known to promote Th17 lymphocyte differentiation [39]. Furthermore, co-culturing adult T lymphocytes with supernatants from human bronchial epithelial cells chronically-infected with the RSV A2 long strain promoted the differentiation of naïve T lymphocytes to Th2 and Th17 lymphocytes but not to Th1 lymphocytes [54]. These studies of T lymphocytes responses during RSV infection indicate that besides Th1 and Th2 responses, Th17 responses also occur. These studies, therefore, suggest that the Th17 response is beneficial in some cases of RSV infection. Th17 responses have, however, also been linked with pathology in the respiratory tract during severe neutrophil dominated asthma. More research is, therefore, needed to unravel the complex consequences of IL-17 production and when this is beneficial, when not and why. 

## 4. The role of Th17 lymphocytes in the patho-physiology of RSV infection: some insights from *in vitro* and animal model studies.

To better understand the role that Th17 lymphocytes play in the patho-physiology of RSV infection animal models and *in vitro* systems have been studied. Different strains of RSV were used to immunize mice and assess the immune response to RSV infection. In addition, cell lines were used to assess the direct effect viral particles have on immune cells. RSV strains “A2” and “A2 long” were used to immunize mice to define the nature of the response including immune cell infiltration during RSV infection. The RSV strain “line 19”, by contrast, was used to study cellular and molecular mechanisms involved in excessive mucus secretion and IL-13 production. Studies using the three strains of RSV revealed that infection induces IL-17 production in mice. However, no studies have been carried out to compare and contrast the responses initiated by the three strains in the same experiment.

### 4.1. Infection with RSV induces IL-17 production which promotes neutrophil influx during the early response.

Immunization of wild-type mice with RSV strain A2 resulted in complement activation by infected epithelial cells leading to the production of C3a which induced tachykinin and Substance P release. These mediators bound to their receptors on T lymphocytes, neutrophils and monocytes and lead to IL-17A production by CD11b^+^ myeloid cells during the early phase of the response [55]. In addition, RSV RNA particles trigger innate immune system-mediated inflammatory responses by binding to TLRs. Studies on the interaction between viral particles and cell lines *in vitro* revealed that IL-17 acted synergistically with RSV RNA particles to induce IL-6 and IL-8 production by fibroblasts. Thus, RSV strain A2 RNA induced an innate immune-like response by binding to TLR3 and this response was enhanced by IL-17 [56]. Furthermore, IL-17 produced during the infection increased the influx of neutrophils. The influx of neutrophils was also observed following infection with the RSV strain A line 19. In this setting neutrophils were recruited by IL-8 which was induced by IL-17 [32] (Figure 1).

The impact of RSV infection on the cooperation between the innate system and IL-17/IL-17 producing cells is, however, complex. Thus, in addition to binding TLR3, single stranded RSV RNA particles bind TLR7 on DCs, plasmacytoid DCs, B lymphocytes and macrophages and induce IL-12 production, which promotes Th1-mediated responses (Figure 1). This can impact the balance between Th1 and Th17 responses and the patho-physiological response *in vivo*. For example, infection of TLR7 deficient mice with RSV Strain A line 19 resulted in increased numbers of Th17 lymphocytes due to an increase in IL-23 production by DCs. This response caused more pathology through the consequent increase in IL-13 and mucus production in TLR7 deficient mice compared with wild-type mice [24]. Furthermore, the inflammatory response to the mouse homologue of RSV, pneumo virus of mice (PVM), was diminished when TLR7 was missing [57]. This is somewhat analogous to the situation in newly born infants in whom effector Th17 lymphocytes are recruited when the activation of Th1 responses is inadequate. 

### 4.2. Activation of Th17 and Th2 cells in response to RSV infection

An efficient immune response to RSV infection is dependent on antigen recognition and presentation by DCs in local lymph nodes. Viral antigen presentation by DCs results in the activation and migration of CD4^+^ and CD8^+^ lymphocytes to the lung. The process of DC migration is dependent on the chemokine CCR7. Kallal and colleagues noted that CCR7 deficient mice had impaired lymph node formation and, instead, responded by activating T lymphocytes in local ectopic lymphoid structures in response to infection with RSV Strain A, line 19. The T lymphocyte response to RSV infection in these structures was dominated by Th17 lymphocytes. These Th17 lymphocytes promoted pathology by inducing the production of IL-13 and IL-21 which induced excessive mucus production [58]. A similar response was observed in the absence of IFNγ signaling in STAT1-deficient mice. Thus, infection of STAT1-deficient mice with RSV strain A2 resulted in elevated IL-13 and IL-17 levels, production of excess mucus and airway inflammation. Just as was the case in TLR7^-/-^ mice, elevated IL-17 levels in STAT1-deficient mice were due to increased production of the IL-23 [25]. In addition, to the inflammatory effects mediated by excess IL-17, it suppressed the ability of CD8^+^ to kill cells infected with RSV [59]. This latter study revealed that Th17-derived IL-17 bound to IL-17RA on CD8^+^ T lymphocytes and impaired their ability to reduce viral load and reduce the number of infected cells in the lung. The role of excess IL-17 in promoting pathology is further supported by the ability of neutralizing anti-IL-17 antibodies to reduce mucus and IL-13 production and increase viral clearance [36] (Table 1 and Figure 1). *In vitro* studies revealed that IL-17 enhanced mucus production by directly upregulating transcription of the mucus gene *MUC5B* in human tracheal and bronchial epithelial cell lines [60,61]. This upregulation of the *MUC5B* gene was shown to be dependent on ERK signalling and the activation NF-κB [60,61]

A number of studies in which mice were infected with RSV particles have shown that the mice simultaneously produced IL-17 and IL-13 suggesting that the Th17 response is concomitant with the Th2 response [25,32,54,58]. The molecular mechanisms that underpin the co-production of IL-13 and IL-17 were further explored in STAT1-deficient mice. Newcomb *et al*. observed that IL-13 produced during infection of mice with RSV Strain A2 was capable, perhaps paradoxically, of suppressing IL-17 production [73]. Using double STAT1- and IL-13-deficient mice for immunization experiments, these investigators observed higher levels of IL-17 production than in mice deficient in STAT1 alone. Increased IL-17 production in STAT1/IL-13-deficient mice was explained by the fact that IL-10 production, which is induced by IL-13, reduces IL-17 production by Th17 lymphocytes. In addition, Th17 lymphocytes have been reported to express IL-13 receptor alpha (IL-13Rα, also known as IL-13RA) suggesting that these cells could be directly modulated by IL-13 [62]. As IL-17 has been shown to increase mucus production through enhancing IL-13 production, these findings may suggest that IL-13 can also negatively regulate Th17 lymphocytes through a negative feedback mechanism [33]. 

### 4.3. IL-17 causes RSV-mediated exacerbation of asthma

IL-13 and mucus production are not only associated with IL-17 production but a feature of virally-exacerbated asthma. In addition, a number of studies have indicated that IL-17 is involved in severe asthma [28,29]. Therefore, the involvement of IL-17 has been explored in animal models of RSV infection concomitant with experimental asthma. For example, infection of mice with RSV Strain A2 subsequent to immunization with ovalbumin (OVA) induced experimental asthma and increased the production of mucus-associated proteins, Muc5ac and Gob-5 [63]. Mice injected with OVA, or with RSV alone, also upregulated the expression of genes encoding the mucus-associated proteins but gene expression persisted for longer periods in the OVA and RSV-immunized mice compared with those immunized with either OVA or RSV alone. Importantly, the increase in mucus production was associated with increased levels of IL-17 in the lungs [63]. Another study in which investigators used cockroach allergen (CRA) with RSV Strain A line 19 to induce asthma in mice provided further evidence for Th17 lymphocytes involvement in experimental asthma. Thus, stimulated T lymphocytes from lymph nodes of RSV/CRA-immunized mice produced IL-17 while mice immunized with CRA alone did not. Furthermore, administration of anti-IL17 antibody intraperitoneally suppressed the expression of Muc5ac and Gob5 in the lung, and IL-13 production in lymph nodes but increased the number of CD8^+^ lymphocytes [32]. These observations are further evidence for a role for IL-17 in viral exacerbation of asthma.

**Table 1:** Responses and products released by the cells present in the respiratory tract when stimulated with IL-17.

Cell type	Response	in vitro/in vivo	Reference
**CD8+ lymphocyte**	Reduced RSV clearance	*in vivo*	[32]
**Epithelial cells**	IL-6, IL-8, PGE_2_	*in vitro*	[64]
	MUC5B, MUC5AC	*in vitro*	[60,61]
	CCL20	*in vitro*	[65]
	beta defensin 2	*in vitro*	[66]
	IL-19 **	*in vitro*	[67]
**Endothelial cells**	IL-6, IL-8, PGE_2_		
**Lung microvascular endothelial cells**	CXCL1 (GROα), CXCL5, and IL-8 *	*in vitro*	[68]
**Fibroblasts**	IL-6, IL-8, PGE_2_	*in vitro*	[64] [69]*
**Smooth muscle**	AHR (OVA induced asthma)	*in vivo*	[70]
	Contraction	*in vitro*	

* IL-17 potentiates the response by IL-1 beta and TNFα. ** IL-17 potentiates the response by IL-13.

## 5. Summary

Th17 lymphocytes are important contributors to both protective immune responses and the pathology associated with RSV infection. The involvement of Th17 cells in the patho-physiology that accompanies RSV infections is of great topical interest. Measurements of IL-17 levels in plasma and BAL fluids from RSV-infected infants have indicated that the cytokine can be beneficial. These studies have also suggested that Th17 responses during RSV infections are independent of Th1 and Th2 responses and that they are, in some infants, supersede an immature/inadequate Th1 immune response in the newly born [39]. Studies of IL-17 in animal models and *in vitro* culture systems have revealed that the lack of INFγ-mediated response enhances Th17 response. Such a response is driven by IL-23 which is produced in preference to IL-12 [24,25]. *In vitro* systems and animal models have showed that IL-17 *per se*, or together with RSV RNA particles, can induce inflammatory cytokine and chemokine responses that promote the influx of neutrophils to sites of infection [32]. As have been shown in other models of inflammation, IL-17 can also orchestrate the development of tertiary lymphoid tissues in the lung [58,71], [72]. These structures are termed inducible bronchus-associated lymphoid tissues and are similar to structures found in RA patients with pulmonary complications (an autoimmune disease associated with IL-17)[73]. In addition to enhancing the inflammatory response that accompanies RSV infections, IL-17 has also been shown to suppress the ability of CD8+ lymphocytes to kill virally-infected cells and reduce the viral load [32]. Furthermore, both *in vitro* and in animal model studies have confirmed that IL-17 enhances mucus production by acting directly on epithelial cells. This mucus production was also shown to be accompanied by IL-13 production which acts synergistically with IL-17 to enhance mucus production. However, high levels of IL-13 were also reported to inhibit IL-17 production. The induction of IL-17 production by RSV infection exacerbates asthma through enhancing mucus production. It is intriguing that IL-17F was not upregulated concomitant with IL-17A in one model of RSV infection [32]. Further studies to determine if IL-17F has a distinct role in RSV infection are warranted. The involvement of other Th17 cytokines, such as IL-21 and IL-22 in RSV patho-physiology also remains to be determined.

Taken together, studies of infected individuals and animal models have revealed that IL-17 can have both beneficial and pathogenic effects during RSV infection. In animal models, just as in cases of patients with asthma, IL-17 induces pathology by enhancing neutrophil influx, mucus and IL-13 production. However, the beneficial effects of IL-17 continue to be debated. It is intriguing to note that although Th17 lymphocytes have evolved together with the rest of the adaptive immune system, the emergence of the IL-17 family of cytokines predates chordates [74]. Th17 lymphocytes differentiate in response to inflammatory cytokines, preferably with low level of TCR engagement and their expansion is favoured by low oxygen levels [21,22]. The cells might, therefore, emerge as a weak alternative to the more efficient antiviral response mediated by a missing, or immature/inadequate Th1 lymphocyte response. Under such circumstances, Th17 lymphocytes may provide a response that straddles adaptive and innate immune responses resulting in mucus release, neutrophil influx and augmentation of local tertiary lymphoid structures. 
